# Ultrasound-based clinical profiles for predicting the risk of intradialytic hypotension in critically ill patients on intermittent dialysis: a prospective observational study

**DOI:** 10.1186/s13054-019-2668-2

**Published:** 2019-12-02

**Authors:** Rogerio da Hora Passos, Juliana Caldas, Joao Gabriel Rosa Ramos, Erica Batista dos Santos Galvão de Melo, Michel Por Deus Ribeiro, Maria Fernanda Coelho Alves, Paulo Benigno Pena Batista, Octavio Henrique Coelho Messeder, Augusto Manoel de Carvalho de Farias, Etienne Macedo, Jean Jacques Rouby

**Affiliations:** 1 Critical Care Unit and Nephrology Department, Hospital Português and Hospital São Rafael, Salvador, Bahia Brazil; 2 Critical Care Unit, Hospital São Rafael, Salvador, Bahia Brazil; 3 Critical Care Unit, Hospital Português, Salvador, Bahia Brazil; 4 Nephrology Department, Hospital São Rafael, Salvador, Bahia Brazil; 5 Critical Care Unit, Hospital Português and Hospital São Rafael, Salvador, Bahia Brazil; 6 Nephrology Department, Hospital Português, Salvador, Bahia Brazil; 7 Critical Care Unit and Nephrology Department, Hospital São Rafael, Salvador, Bahia Brazil; 8 Division of Nephrology, Department of Medicine, University of California, San Diego, USA; 9 Multidisciplinary Intensive Care Unit, Department of Anesthesiology and Critical Care Medicine, La Pitié-Salpêtrière Hospital, Assistance Publique Hôpitaux de Paris, Sorbonne University of Paris, Paris, France

**Keywords:** Ultrasound, Dialysis, Hypotension, Acute kidney injury, Critically ill patients, Profiles

## Abstract

**Background:**

Intradialytic hypotension, a complication of intermittent hemodialysis, decreases the efficacy of dialysis and increases long-term mortality. This study was aimed to determine whether different predialysis ultrasound cardiopulmonary profiles could predict intradialytic hypotension.

**Methods:**

This prospective observational single-center study was performed in 248 critically ill patients with acute kidney injury undergoing intermittent hemodialysis. Immediately before hemodialysis, vena cava collapsibility was measured by vena cava ultrasound and pulmonary congestion by lung ultrasound. Factors predicting intradialytic hypotension were identified by multiple logistic regression analysis.

**Results:**

Intradialytic hypotension was observed in 31.9% (*n* = 79) of the patients, interruption of dialysis because of intradialytic hypotension occurred in 6.8% (*n* = 31) of the sessions, and overall 28-day mortality was 20.1% (*n* = 50). Patients were classified in four ultrasound profiles: (A) 108 with B lines > 14 and vena cava collapsibility > 11.5 mm m^−2^, (B) 38 with B lines < 14 and vena cava collapsibility ≤ 11.5 mm m^−2^, (C) 36 with B lines > 14 and vena cava collapsibility Di ≤ 11.5 mm m^−2^, and (D) 66 with B lines < 14 and vena cava collapsibility > 11.5 mm m^−2^. There was an increased risk of intradialytic hypotension in patients receiving norepinephrine (odds ratios = 15, *p* = 0.001) and with profiles B (odds ratios = 12, *p* = 0.001) and C (odds ratios = 17, *p* = 0.001).

**Conclusion:**

In critically ill patients on intermittent hemodialysis, the absence of hypervolemia as assessed by lung and vena cava ultrasound predisposes to intradialytic hypotension and suggests alternative techniques of hemodialysis to provide better hemodynamic stability.

## Introduction

Ultrafiltration-induced fluid removal for fluid balance control is a major target of renal replacement therapy [1]. However, in critically ill patients, intradialytic hypotension (IDH) is a frequent complication of intermittent hemodialysis and it may decrease the efficacy of renal replacement therapy [2, 3].

The ultrafiltration rate is set to ensure fluid removal required to reduce fluid overload. However, assessment of fluid overload in critically ill patients may be a challenge, because pulmonary congestion is poorly correlated with clinical signs [4]. One alternative approach is the utilization of transthoracic lung ultrasound. A score based on the number of B lines accurately measures the degree of lung congestion and may guide ultrafiltration rate [4–6]. In addition, the rate of disappearance of B lines during intermittent hemodialysis shows a good correlation with the volume of ultrafiltration and dry weight [7, 8].

The pathogenesis of IDH includes the dialysis process itself and critically ill patient-related factors. It has been postulated that the ultrafiltration rate may lead to a reduction in preload, predisposing to hemodynamic instability. Moreover, the dialysis process may interfere with compensatory mechanisms, predisposing to an increased risk of hypotension [9]. As such, it is not clear whether IDH is directly related to preload dependence or to other factors. Clinical studies have been controversial. One study has reported that a positive passive leg raising test before starting dialysis predicts IDH during renal replacement therapy [10]. Another has shown that, in critically ill patients, the majority of hypotensive episodes occurring during intermittent hemodialysis are unrelated to preload dependence and likely related to vasomotor tone alterations [11]. Furthermore, clinical and radiological tools for assessing volume status are subjected to a wide variability of interpretation [12, 13]. As an alternative, the ultrasound measurement of inferior vena cava diameter has been reported to be an accurate method for the assessment of fluid status in critically ill patients [14] and in patients undergoing hemodialysis [15–17].

Although several studies have confirmed the emergent role of biomarkers such as natriuretic peptides [18], copeptin, and bioimpedance vector analysis (BIVA) in the management of volume overload, bedside lung ultrasound appears a sensitive tool for evaluating changes in extravascular lung water in dialysis patients [18, 19]. The primary aim of the present study is to determine whether different predialytic cardiopulmonary profiles, defined on sonographic findings, could predict IDH in critically ill patients undergoing intermittent hemodialysis. The secondary aim was to identify risk factors for IDH in this population.

## Methods

### Study design

This was a prospective observational single-center study performed between January 1, 2015, and April 30, 2018, in a 30-bed medical intensive care unit (ICU), at Hospital Português, a tertiary hospital in Salvador, Brazil. The study was approved by the Ethical Committee from Centro de Estudos Egaz Muniz (CAAE: 89428318.000005029). Written informed consent was waived for this observational and non-interventional study. Critically ill patients were included in the study if they fulfilled the following criteria: (i) age > 18 years, (ii) acute kidney injury (AKI) defined by KDIGO 3, and (iii) treatment by intermittent hemodialysis. Patients with right ventricle dysfunction, valvar heart disease, lung hyperinflation, increased abdominal pressure, marked inferior vena cava respiratory translational motion, and the use of compression stockings and patients with inadequate transthoracic window were excluded from the study.

### Intermittent hemodialysis sessions

The nephrology team in charge of patient care was responsible for the timing of initiation of dialysis and prescription. Intermittent hemodialysis sessions were performed on the basis of standard clinical guidelines, including AKI with hemodynamic stability, ongoing hypercatabolism, hyperkalemia, severe acidosis, presumed volume overload, and respiratory distress. The indication for intermittent hemodialysis refers to patients without vasopressors or in low dose of vasopressors (norepinephrine dose ≤ 0.3 mg kg^−1^ min^−1^) for at least 6 h before initiation of dialysis with mean arterial pressure (MAP) ≥ 65 mmHg. For each patient, clinical, laboratory, and hemodynamic variables were used to inform clinical decision-making of ultrafiltration rate. Intermittent hemodialysis was performed with Fresenius 4008 S (Gambro Hospal, Meyzieu, France), and dialysate concentrate solutions with 1.75 mmol/L calcium concentration. Intradialytic hypotension (IDH) was defined as the occurrence of a MAP below 65 mmHg during the dialysis session [11].

### Ultrasound procedures and classifications

Vena cava collapsibility measurement and B line determination were performed by physicians with expertise in cardiac and lung ultrasound for critically ill patients [7, 20–22] (see Additional file 5).

### Statistical analysis

Statistical analysis was performed using SPSS version 19.0 for Windows (SPSS Inc., Chicago, IL). Data were tested for normality by visual inspection and the use of Kolmogorov-Smirnov test. Continuous variables were expressed as median (interquartile range, IQR) and were compared by a nonparametric Mann-Whitney test. Results are expressed as mean ± SD or median (IQR). Proportions were compared by the *χ*^2^ test. Variables were compared between groups of sessions (with hypotension vs without hypotension). The binary classification (“no intradialytic hypotension” vs “intradialytic hypotension”) was used as an outcome variable in a way that “no hypotension” and “hypotension” were coded as 0 and 1, respectively. Quantitative and qualitative variables associated with hypotension with a *p* value below 0.05 in univariate analysis were selected for inclusion in a multivariable logistic regression model. Logistic regression with both categorical and continuous independent variables was used to build predictive models for the occurrence of hypotension. A one way-ANOVA was used for comparison between groups of patients according to ultrasound profiles. The four “ultrasound profiles” were compared for association with hypotension, dialysis discontinuation, or mortality in 28 days, using *χ*^2^ analysis. Statistical significance was assumed at the 5% level.

## Results

From January 2016 to March 2018, 248 AKI patients requiring intermittent hemodialysis were considered eligible. Median age was 68, and the age range was 58–76; 149 (60.1%) patients were male; the median Charlson score was 10 (8–12); the median APACHE II score was 15 (12–18); and SOFA score was 8 (6–10).

### Intradialytic hypotension: incidence and predictive factors

Patients’ characteristics are shown in Table 1 and hemodialysis sessions’ characteristics in Table 2. IDH was observed in 31.9% (*n* = 79) of the patients, interruption of dialysis because of IDH occurred in 6.8% (*n* = 31) of the sessions, and overall 28-day mortality was 20.1%. The presence of sepsis, the use of norepinephrine, a low predialysis MAP, a high predialysis lactate level, the use of mechanical ventilation, and elderly age were factors significantly associated with IDH (Table 1). There was a difference in median ultrafiltration volume (Table 1) between patients with and without IDH: 893 ml (0–1500) in patients with IDH vs 1242 ml (675–2000) in patients without IDH (*p* = 0.020).

**Table 1:** Clinical, ultrasound, and biological characteristics of patients

	All patients	Intradialytic hypotension		*p*
		No	Yes	
	*N* = 248	169 (68.1%)	79 (31.9%)	
Clinical characteristics				
Gender				0.684
- Male, *n* (%)	149 (60.1%)	103 (60.9%)	46 (58.2%)	
- Female, *n* (%)	99 (39.9%)	66 (39.1%)	33 (41.8%)	
Age (years)	68.0 (58.2–76)	66 (55–76)	70 (64–76)	0.018
Charlson score	10 (8–12)	10 (8–12)	10 (8–12)	0.116
APACHE II	15 (12–18)	14 (12–18)	16 (13–18)	0.215
SOFA	8 (6–100)	8 (6–10)	8 (7–10)	0.057
Sepsis, *n* (%)	123 (49.6%)	71 (42%)	52 (65.8%)	< 0.001
Use of norepinephrine, *n* (%)	37 (14.9%)	5 (3%)	32 (40.5%)	< 0.001
Mechanical ventilation, *n* (%)	34 (13.7%)	14 (8.3%)	20 (25.3%)	< 0.001
Predialytic arterial pressure				
Systolic blood pressure, mmHg	132 (114.2–152)	140 (123–160)	114 (103–135)	< 0.001
Diastolic blood pressure, mmHg	70 (60.2–80.7)	71 (64–86)	64 (58–73)	< 0.001
Mean blood pressure, mmHg	89.5 (79.0–105.0)	94 (84–109)	81 (75–90)	< 0.001
Biological characteristics				
Hemoglobin, g/dL	9 (7.6–10.1)	9 (7.4–10.1)	9 (7.8–10.2)	0.839
Bicarbonate, mEq/L	20(18–23)	20 (18–23)	20 (16–22)	0.321
Sodium, mEq/L	138 (138–140)	138 (135–141)	139 (134–141)	0.565
Urea, mg/dL	143 (114.2–194)	143 (110–195)	142 (119–194)	0.659
Lactate, mmol/L	1.4 (1.1–1.9)	1.3 (1.1–1.7)	1.7 (1.2–2.2)	< 0.001
Fluid balance, mL	1772 (1540–2320)	1800 (1535–2320)	1750 (1540–2350)	0.762
Ultrasound characteristics				
Pulmonary congestion, *n* (%)	144 (58.1%)	106 (62.7%)	38 (48.1%)	0.030
IVC collapsibility, *n* (%)	74 (29.8%)	14 (8.3%)	60 (75.9%)	< 0.001

*APACHE* Acute Physiology, Age, Chronic Health Evaluation; *SOFA* Sequential Organ Failure Assessment score; *IVC* inferior vena cava; *IQR* interquartile. Data are expressed as number (percentage) for categorical variables or median (1st quartile–3rd quartile) for continuous variables

**Table 2:** Dialysis sessions’ characteristics

Dialysis characteristics	All patients	Intradialytic hypotension		*p*
		No	Yes	
	*N* = 248	169 (68.1%)	79 (31.9%)	
Duration, min	240 (180–240)	240 (180–240)	210 (150–240)	0.140
Ultrafiltration, mL	1000 (200–2000)	1.242.01	893.03	0.020
Blood flow, mL/min	300 (250–300)			0.010
- 200		24 (14.2%)	24 (30.4%)	
- 250		47 (27.8%)	16 (20.3%)	
- 300		98 (58%)	38 (48.1%)	
- 350		0 (0%)	1 (1.3%)	
Dialysate flow, mL/min	500 (500–500)			0.002
- 300		12 (7.1%)	17 (21.5%)	
- 320		0 (0%)	1 (1.3%)	
- 500		157 (92.9%)	61 (77.2%)	
Temperature, °C	36 (36–36)	36.0 (35.5–36.5)	36.0 (35.5–36.5)	0.416
Sodium, mEq/L	138 (138–140)	138 (138–140)	138 (135–141)	

Data are expressed as number (percentage) for categorical variables or median (1st quartile–3rd quartile) for continuous variables

Regarding ultrasound findings, pulmonary congestion (as defined by a B line score ≥ 14) was found in 38 (48.1%) patients with IDH and 106 (62.7%) patients without IDH (*p* = 0.030). Inferior vena cava collapsibility (as defined by a VCDi ≤ 11.5 mm m^2^) was found in 60 (75.9%) patients with IDH and 14 (8.3%) patients without IDH (*p* < 0.001).

The predictive multiple logistic regression model of IDH demonstrated that MAP, use of norepinephrine, and inferior vena cava collapsibility were associated with the occurrence of IDH (Table 3). Specifically, pulmonary congestion (a B line score ≥ 14) and higher MAP appeared protective against IDH (Table 3).

**Table 3:** Multivariate logistic regression analysis for the occurrence of intradialytic hypotension

Variable	Parameter estimated	Standard error	Odds ratio	95% CI	*p*
Age	0.018	0.015	1.018	0.989–1.049	0.228
Blood flow rate	0.006	0.007	1.006	0.993–1.019	0.379
Dialysate flow rate	− 0.007	0.004	0.993	0.984–1.001	0.096
Ultrafiltration	0.001	0.004	1.000	1.000–1.001	0.136
Mean blood pressure	− 0.039	0.015	0.962	0.934–0.991	0.010
Lactate	− 0.187	0.398	0.829	0.380–1.810	0.638
Use of norepinephrine	2.736	0.679	15.425	4.078–58.353	0.001
Pulmonary congestion	− 1.025	0.462	0.448	0.106–0.888	0.027
Inferior vena cava collapsibility	3.573	0.487	35.635	13.722–92.538	0.001
Mechanical ventilation	− 0.803	0.737	0.359	0.106–1.900	0.276
Sepsis	0.584	0.466	1.793	0.719–4.470	0.210
Constant	1.825				

### Ultrasound profiles

**Fig. 1:**
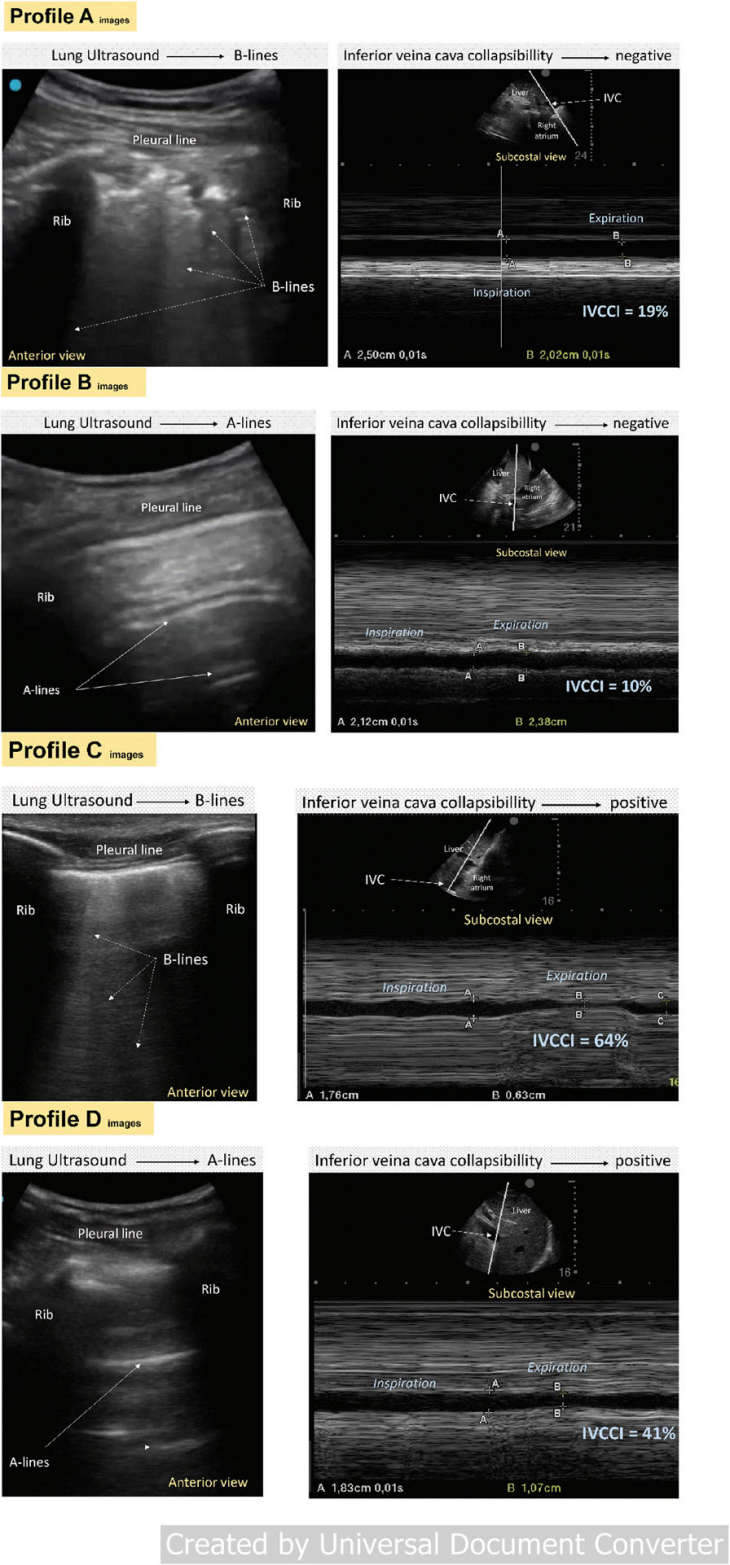
Vena cava and lung ultrasound profiles. (Profile A) 66-year-old patient admitted to the intensive care unit for community-acquired pneumonia with acute kidney injury KDIGO 3 requiring hemodialysis. The patient had a positive fluid balance of 6.5 L and did not receive vasoactive support. In the right upper anterior thoracic region, multiple coalescent B lines issued from justapleural consolidations typical of bronchopneumonia are visible. As shown in the corresponding video file (Additional files 1, 2, 3, and 4), lung sliding is nearly abolished caused by inflammation/infection of pleural layers. The vena cava appears well filled and does not show any significant collapsibility with respiratory movements. (Profile B) 28-year-old patient admitted to the intensive care unit for a urinary tract infection, and acute kidney injury KDIGO 3 requiring dialysis was diagnosed. The patient had a positive fluid balance of 2.5 L, and he was on vasoactive support. In the right upper anterior thoracic region, predominant A lines are visible. As shown in the corresponding video file (Additional files 1, 2, 3, and 4), lung sliding is normal. The vena cava appears filled and shows not significant collapsibility with respiratory movements. (Profile C) 53-year-old patient admitted to the intensive care unit for an alcoholic acute pancreatitis. AKI (KDIGO) 2 was diagnosed, and intermittent dialysis was initiated. The patient had a positive fluid balance of 8.5 L, and he was on vasoactive support. In the right upper anterior thoracic region, white lines from the pleural line to the bottom (B–lines–comets) are visible. As shown in the corresponding video file (Additional files 1, 2, 3 and 4), lung sliding is normal. The vena cava appears collapsed with respiratory movements. (Profile D) 73-year-old patient admitted to the intensive care unit for an acute mesenteric ischemia treated by an extended right hemicolectomy. The patient developed AKI KDIGO 3, and intermittent dialysis was started. The patient had a positive fluid balance of 5.5 L and did not receive vasoactive support. In the right upper anterior thoracic region, horizontal lines (A lines) are visible. As shown in the corresponding video file (Additional files 1, 2, 3, and 4), lung sliding is normal. The vena cava appears collapsed with respiratory movements

Based on lung ultrasound and VCDi, we could classify patients in four distinct profiles (Fig. 1) (Additional files 1, 2, 3, and 4): (A) 108 patients had pulmonary congestion and hypervolemia, (B) 38 patients did not have pulmonary congestion nor hypervolemia, (C) 36 patients had pulmonary congestion without hypervolemia, and (D) 66 patients had hypervolemia without pulmonary congestion. Baseline characteristics of the patients in the four ultrasound profiles are listed in Table 4. The presence of IDH was different between profiles (*p* < 0.001). All other variables but age were similar between the four different profiles.

**Table 4:** Baseline characteristics of patients according to the ultrasound profile

Profile	A, *n* = 108	B, *n* = 38	C, *n* = 36	D, *n* = 66	*p*
Hypotension, *n* (%)	8 (7.4%)	30 (78.9%)	30 (83.3%)	11 (16.7%)	0.001
Gender					0.346
Male, *n* (%)	67 (62%)	26 (68.4%)	22 (14.8%)	34 (51.4%)	
Female, *n* (%)	41 (41.4%)	12 (31.6%)	14 (38.9%)	32 (48.5%)	
Age (years)	65.0 ± 15.2	69.9 ± 11.8	66.0 ± 12.4	66.0 ± 18.1	0.016
Charlson score	10.3 ± 2.7	10.6 ± 2.8	10.7 ± 2.6	10.0 ± 2.8	0.113
APACHE II	15.2 ± 3.7	15.6 ± 4.3	15.9 ± 3.3	15.4 ± 5.6	0.470
SOFA	8.2 ± 2.3	8.4 ± 2.6	9.2 ± 2.6	8.4 ± 2.7	0.677
Sepsis, *n* (%)	59 (54.6%)	17 (44.7%)	15 (41.7%)	32 (48.5%)	0.495
Use of norepinephrine, *n* (%)	19 (17.6%)	6 (15.8%)	4 (11.1%)	8 (12.1%)	0.694
Mechanical ventilation, *n* (%)	16 (14.8%)	8 (21.1%)	3 (8.3%)	7 (10.6%)	0.353
Fluid balance (milliliters)	1952.2 ± 702.0	1953.0 ± 673.5	1899.8 ± 667.5	1951.8 ± 592.9	0.469
Mortality 28 days, *n* (%)	19 (17.6%)	6 (15.8%)	5 (13.9%)	20 (30.3%)	0.112
Dialysis data					
Duration, min	212.4 ± 47.5	202.6 ± 53.1	195.14 ± 81.1	212.7 ± 40.7	0.958
Ultrafiltration, mL/min	1197.2 ± 836.6	1072.4 ± 923.3	813.9 ± 818	1228.8 ± 40.7	0.428
Blood flow rate, mL/min	273.15 ± 35.80	248.7 ± 48.6	262.5 ± 42.0	274.24 ± 35.3	0.420
Dialysate flow rate, mL/min	485.18 ± 56.62	447.9 ± 88.4	455.5 ± 84.3	487.9 ± 48.1	0.628
Sodium, mEq/L	138.66 ± 1.28	138.6 ± 0.9	138.8 ± 1.5	138.8 ± 1.9	0.841
Systemic data					
Temperature	36.06 ± 0.25	36.0 ± 0.3	36.0 ± 0.3	36.0 ± 26.1	0.326
Systolic blood pressure, mmHg	139.1 ± 28.5	126.0 ± 0.3	123.0 ± 22.5	144.4 ± 26.1	0.855
Diastolic blood pressure, mmHg	72.8 ± 15.8	68.6 ± 16.9	67.2 ± 12.7	73.9 ± 16.8	0.831
Mean blood pressure, mmHg	94.9 ± 18.4	87.8 ± 18.3	85.8 ± 13.6	97.4 ± 17.5	0.793
Blood test data					
Hemoglobin, /dL	9.1 ± 2.2	9.2 ± 2.2	8.6 ± 2.2	8.6 ± 2.2	0.262
Bicarbonate, mEq/L	19.7 ± 3.8	18.8 ± 3.8	20.1 ± 5.2	20.1 ± 5.2	0.767
Sodium, mEq/L	138.2 ± 4.5	138.9 ± 8.5	137.4 ± 5.4	137.0 ± 6.6	0.396
Urea, mg/dL	157.6 ± 59.3	159.9 ± 50.3	157.5 ± 47.8	150.1 ± 61.7	0.802
Lactate, mmol/L	1.5 ± 0.6	1.8 ± 0.85	2.0 ± 1.1	1.5 ± 0.5	0.242

*SOFA* Sequential Organ Failure Assessment, *APACHE* Acute Physiology and Chronic Health Evaluation. Data are expressed as mean ± standard deviation

### Clinical outcomes vs ultrasound profiles

Table 5 shows the risk for IDH, early interruption of intermittent hemodialysis, and 28-day mortality. Patients in profile D appeared to have a lower risk for all outcomes. There was an increased risk of IDH, dialysis discontinuation, and 28-day mortality in patients with profiles B and C.

**Table 5:** Ultrasound profiles vs outcomes

Profile	Parameter estimated	Standard error	Odds ratio	95% CI		*p*
Hypotension						
A	− 2.530	0.404	0.08	0.036	0.176	0.001
B	2.511	0.430	12.321	5.304	28.624	0.001
C	2.811	0.476	16.633	6.544	42.277	0.001
D	− 1.093	0.364	0.335	0.164	0.684	0.003
Dialysis discontinuation						
A	− 2.608	1.039	0.074	0.010	0.565	0.012
B	4.516	0.526	4.516	1.601	12.743	0.004
C	1.863	0.526	6.444	2.298	18.071	0.001
D	− 1.835	1.041	0.160	0.021	1.228	0.780
Mortality 28 days						
A	− 1.072	0.360	0.342	0.169	0.694	0.003
B	0.580	0.399	1.786	0.817	3.907	0.146
C	1.284	0.385	3.612	1.697	7.688	0.001
D	− 0.172	0.368	0.842	0.410	1.731	0.640

## Discussion

In this study, we describe four clinical profiles based on ultrasound findings and their relation to IDH, interruption of dialysis, and overall mortality at 28 days. Patients with pulmonary congestion and hypervolemia (profile A) had a lower risk of IDH. In contrast, patients without pulmonary congestion and hypervolemia had the highest incidence of IDH. Sepsis, the use of norepinephrine, a low predialysis MAP, lactate level, and mechanical ventilation were significantly associated with IDH.

The pathogenesis of IDH includes the dialysis process itself and critically ill patient-related factors such as sepsis and sedation. Cardiac output and peripheral vasomotor tone are the main determinants of arterial pressure. In critically ill patients, dialysis hypotension is a result of the imbalance of these two variables [23]. Sepsis is the main etiology of AKI and causes a decrease in the peripheral vasomotor tone, induced by the release of vasodilating inflammatory mediators [24, 25]. During the ultrafiltration process, plasma volume decreases leading to an increase in protein level [26]. The resulting increase in oncotic pressure induces a water shift from interstitial and intracellular compartments toward intravascular compartment, which limits ultrafiltration-induced hypovolemia [27]. When ultrafiltration rate surpasses refilling rate, reduction in preload induces a fall in stroke volume that predisposes to hemodynamic instability [23, 27]. Normally, two physiological mechanisms are activated to maintain cardiac output and arterial pressure despite the reduction of stroke volume: an increase in heart rate and an increase in systemic vascular resistance. The dialysis process, however, interferes with this compensatory process [28]. Several mechanisms have been incriminated [27]: diffusion-induced changes in osmolality impairing baroreceptor activation, dialysis of plasma norepinephrine, calcium dialysate concentrations, and dialysate temperature. The hemodynamic impact of ultrafiltration is higher in critically ill patients with preload dependence and altered vasomotor tone [29].

Application of ultrasound has improved the care of critically ill patients with kidney diseases by providing an accurate estimation of volume status [14, 15] and pulmonary congestion [22]. It is also an accurate monitoring of treatment efficiency: the rate of disappearance of B lines following hemodialysis correlates with ultrafiltration and dry weight [5, 8]. Ultrasound determination of IVC expiratory diameter and collapsibility combined with clinical parameters have been used to monitor volume unloading during hemodialysis [29–32]. In the present study, critically ill patients treated by norepinephrine without hypervolemia were at high risk of IDH. We identified two profiles based on lung and vascular ultrasound able to predict the risk of IDH and guide the type of dialysis session. Critically ill patients with VCDi ≤ 11.5 mm m^−2^, an ultrasound value ruling out hypervolemia, were at risk of IDH in the presence or absence of lung congestion. At the opposite, hypervolemia defined as a VCDi ≥ 11.5 mm m^−2^ was protective against IDH in the presence or absence of lung congestion.

Before starting intermittent hemodialysis, the identification of patients with cardiopulmonary profiles increasing the risk of intradialytic hypotension should incite the clinician to select an alternative dialysis technique. Implementation of practice guidelines for intermittent hemodialysis can lessen hemodynamic instability [2]. Continuous renal replacement therapies (CRRT) provide hemodynamic stability, but the continuous session lasting 24–96 h requires expertise, anticoagulation, and alarm vigilance [33]. Extended daily dialysis is associated with similar outcomes to CRRT; however, further high-quality randomized controlled trials are desirable [34]. A technique of dialysis personalized to the risk of intradialytic hypotension is of critical importance, since intradialytic hypotension can increase mortality [35]. Recently, it has been shown that a high ultrafiltration rate (> 25 ml/kg/day) over the period of RRT significantly reduces the risk of 1-year mortality in 1075 patients with AKI and fluid overload > 5% of body weight [36]. Interestingly, MAP was lower for duration of RRT and cumulative norepinephrine dose higher in patients treated with low or moderate ultrafiltration rate, suggesting that IDH increased the mortality risk. However, it was impossible to conclude whether tolerating intensive ultrafiltration is simply a marker of recovery or a mediator [37]. Our results are similar: it is likely that the increased 28-day mortality observed in the 79 critically ill patients who experienced intradialytic hypotension was partly due to the hypotensive episodes, but the finding of increased mortality in patients with dialytic hypotension does not necessarily mean that hypotension causes mortality.

Our study has some limitations. It is a single-center study, and our findings have to be reproduced and our hypothesis tested in multicenter randomized controlled trials. We have only performed ultrasound studies just before hemodialysis initiation. The vena cava collapsibility was assessed either in patients with or without mechanical ventilation. The patients on mechanical ventilation, however, were lightly sedated and partly spontaneous breathing.

## Conclusion

In critically ill patients with objective indications of emergent initiation of hemodialysis, the absence of hypervolemia with or without pulmonary congestion as assessed by vena cava and lung ultrasound predisposes to intradialytic hypotension. Further studies should be considered to validate these results and personalize the techniques of dialysis according to the risk of intradialytic hypotension.

## Supplementary information

**Additional file 1.** Profile A. Patient with B lines > 14 and VCDi > 11.5 mm.m^− 2^.

**Additional file 2.** Patient with B lines < 14 and VCDi ≤11.5 mm.m^− 2^.

**Additional file 3.** Patient with B lines > 14 and VCDi ≤11.5 mm.m^− 2^.

**Additional file 4.** Patient with B lines < 14 and VCDi > 11.5 mm.m^− 2^.

**Additional file 5.** Ultrasound procedures and classifications

## Data Availability

The datasets used and/or analyzed during the current study are available from the corresponding author on reasonable request.

## Abbreviations

IDH: Intradialytic hypotension
ICU: Intensive care unit
AKI: Acute kidney injury
MAP: Mean arterial pressure
IVC: Inferior vena cava
IVC_max_: Maximal IVC diameter
IVC_min_: Minimum IVC diameter
IVCCI: IVC collapsibility index
IQR: Interquartile range
CRRT: Continuous renal replacement therapies

## References

[1] Claure-Del Granado R, Mehta RL (2016). Fluid overload in the ICU: evaluation and management. BMC Nephrol.

[2] Schortgen F, Soubrier N, Delclaux C, Thuong M, Girou E, Brun-Buisson C (2000). Hemodynamic tolerance of intermittent hemodialysis in critically ill patients: usefulness of practice guidelines. Am J Respir Crit Care Med.

[3] Doshi M, Murray PT (2003). Approach to intradialytic hypotension in intensive care unit patients with acute renal failure. Artif Organs.

[4] Torino C, Gargani L, Sicari R, Letachowicz K, Ekart R, Fliser D (2016). The agreement between auscultation and lung ultrasound in hemodialysis patients: the LUST study. Clin J Am Soc Nephrol CJASN..

[5] Ross DW, Abbasi MM, Jhaveri KD, Sachdeva M, Miller I, Barnett R (2018). Lung ultrasonography in end-stage renal disease: moving from evidence to practice-a narrative review. Clin Kidney J.

[6] Zoccali C, Torino C, Tripepi R, Tripepi G, D’Arrigo G, Postorino M (2013). Pulmonary congestion predicts cardiac events and mortality in ESRD. J Am Soc Nephrol JASN..

[7] Mallamaci F, Benedetto FA, Tripepi R, Rastelli S, Castellino P, Tripepi G (2010). Detection of pulmonary congestion by chest ultrasound in dialysis patients. JACC Cardiovasc Imaging.

[8] Noble VE, Murray AF, Capp R, Sylvia-Reardon MH, Steele DJR, Liteplo A (2009). Ultrasound assessment for extravascular lung water in patients undergoing hemodialysis. Time course for resolution. Chest..

[9] Douvris Adrianna, Zeid Khalid, Hiremath Swapnil, Bagshaw Sean M., Wald Ron, Beaubien-Souligny William, Kong Jennifer, Ronco Claudio, Clark Edward G. (2019). Mechanisms for hemodynamic instability related to renal replacement therapy: a narrative review. Intensive Care Medicine.

[10] Monnet X, Cipriani F, Camous L, Sentenac P, Dres M, Krastinova E (2016). The passive leg raising test to guide fluid removal in critically ill patients. Ann Intensive Care.

[11] Bitker L, Bayle F, Yonis H, Gobert F, Leray V, Taponnier R (2016). Prevalence and risk factors of hypotension associated with preload-dependence during intermittent hemodialysis in critically ill patients. Crit Care.

[12] O’Connor ME, Jones SL, Glassford NJ, Bellomo R, Prowle JR (2017). Defining fluid removal in the intensive care unit: a national and international survey of critical care practice. J Intensive Care Soc.

[13] van der Sande FM, Dekker MJ, Leunissen KML, Kooman JP (2018). Novel insights into the pathogenesis and prevention of intradialytic hypotension. Blood Purif.

[14] Kalantari K, Chang JN, Ronco C, Rosner MH (2013). Assessment of intravascular volume status and volume responsiveness in critically ill patients. Kidney Int.

[15] Niyyar VD, O’Neill WC (2018). Point-of-care ultrasound in the practice of nephrology. Kidney Int.

[16] Krause I, Birk E, Davidovits M, Cleper R, Blieden L, Pinhas L (2001). Inferior vena cava diameter: a useful method for estimation of fluid status in children on haemodialysis. Nephrol Dial Transplant.

[17] Kaptein MJ, Kaptein JS, Oo Z, Kaptein EM (2018). Relationship of inferior vena cava collapsibility to ultrafiltration volume achieved in critically ill hemodialysis patients. Int J Nephrol Renov Dis.

[18] Clerico A, Vittorini S, Passino C (2011). Measurement of the pro-hormone of brain type natriuretic peptide (proBNP): methodological considerations and pathophysiological relevance. Clin Chem Lab Med.

[19] Samoni S, Vigo V, Reséndiz LIB, Villa G, De Rosa S, Nalesso F (2016). Impact of hyperhydration on the mortality risk in critically ill patients admitted in intensive care units: comparison between bioelectrical impedance vector analysis and cumulative fluid balance recording. Crit Care.

[20] Cheriex EC, Leunissen KM, Janssen JH, Mooy JM, van Hooff JP (1989). Echography of the inferior vena cava is a simple and reliable tool for estimation of “dry weight” in haemodialysis patients. Nephrol Dial Transplant.

[21] Muniz Pazeli J, Fagundes Vidigal D, Cestari Grossi T, Silva Fernandes NM, Colugnati F, Baumgratz de Paula R (2014). Can nephrologists use ultrasound to evaluate the inferior vena cava? A cross-sectional study of the agreement between a nephrologist and a cardiologist. Nephron Extra.

[22] Gargani L (2011). Lung ultrasound: a new tool for the cardiologist. Cardiovasc Ultrasound.

[23] Schortgen F (2003). Hypotension during intermittent hemodialysis: new insights into an old problem. Intensive Care Med.

[24] Monge García MI, Guijo González P, Gracia Romero M, Gil Cano A, Oscier C, Rhodes A (2015). Effects of fluid administration on arterial load in septic shock patients. Intensive Care Med.

[25] Ait-Oufella H, Maury E, Lehoux S, Guidet B, Offenstadt G (2010). The endothelium: physiological functions and role in microcirculatory failure during severe sepsis. Intensive Care Med.

[26] Pietribiasi M, Katzarski K, Galach M, Stachowska-Piętka J, Schneditz D, Lindholm B (2015). Kinetics of plasma refilling during hemodialysis sessions with different initial fluid status. ASAIO J Am Soc Artif Intern Organs.

[27] Rouby JJ, Rottembourg J, Durande JP, Basset JY, Legrain M (1978). Importance of the plasma refilling rate in the genesis of hypovolaemic hypotension during regular dialysis and controlled sequential ultrafiltration-haemodialysis. Proc Eur Dial Transpl.

[28] Rouby JJ, Rottembourg J, Durande J-P, Basset J-Y, Degoulet P, Glaser P (1980). Hemodynamic changes induced by regular hemodialysis and sequential ultrafiltration hemodialysis: a comparative study. Kidney Int.

[29] Ronco C, Ricci Z (2008). Renal replacement therapies: physiological review. Intensive Care Med.

[30] Tetsuka T, Ando Y, Ono S, Asano Y (1995). Change in inferior vena caval diameter detected by ultrasonography during and after hemodialysis. ASAIO J Am Soc Artif Intern Organs.

[31] Brennan JM, Ronan A, Goonewardena S, Blair JEA, Hammes M, Shah D (2006). Handcarried ultrasound measurement of the inferior vena cava for assessment of intravascular volume status in the outpatient hemodialysis clinic. Clin J Am Soc Nephrol CJASN.

[32] Guiotto G, Masarone M, Paladino F, Ruggiero E, Scott S, Verde S (2010). Inferior vena cava collapsibility to guide fluid removal in slow continuous ultrafiltration: a pilot study. Intensive Care Med.

[33] Wehle B, Asaba H, Castenfors J, Fürst P, Gunnarsson B, Shaldon S (1979). Hemodynamic changes during sequential ultrafiltration and dialysis. Kidney Int.

[34] Villa G, Neri M, Bellomo R, Cerda J, De Gaudio AR, De Rosa S (2016). Nomenclature for renal replacement therapy and blood purification techniques in critically ill patients: practical applications. Crit Care.

[35] Flythe JE, Xue H, Lynch KE, Curhan GC, Brunelli SM (2015). Association of mortality risk with various definitions of intradialytic hypotension. J Am Soc Nephrol JASN.

[36] Murugan R, Kerti SJ, Chang C-CH, Gallagher M, Clermont G, Palevsky PM (2019). Association of net ultrafiltration rate with mortality among critically ill adults with acute kidney injury receiving continuous venovenous hemodiafiltration: a secondary analysis of the randomized evaluation of normal vs augmented level (RENAL) of renal replacement therapy trial. JAMA Netw Open.

[37] Murugan R, Balakumar V, Kerti SJ, Priyanka P, Chang C-CH, Clermont G (2018). Net ultrafiltration intensity and mortality in critically ill patients with fluid overload. Crit Care.

