# Supplementary material for: Ultrasound-based clinical profiles for predicting the risk of intradialytic hypotension in critically ill patients on intermittent dialysis: a prospective observational study
Source: Crit Care. 2019 Dec 2;23:389. doi: 10.1186/s13054-019-2668-2 (PMC6889608; doi:10.1186/s13054-019-2668-2)
Supplement: Supplementary file 1 — Additional file 1. Profile A. Patient with B lines > 14 and VCDi > 11.5 mm.m− 2. [file 13054_2019_2668_MOESM1_ESM.pptx]

## Slide 1
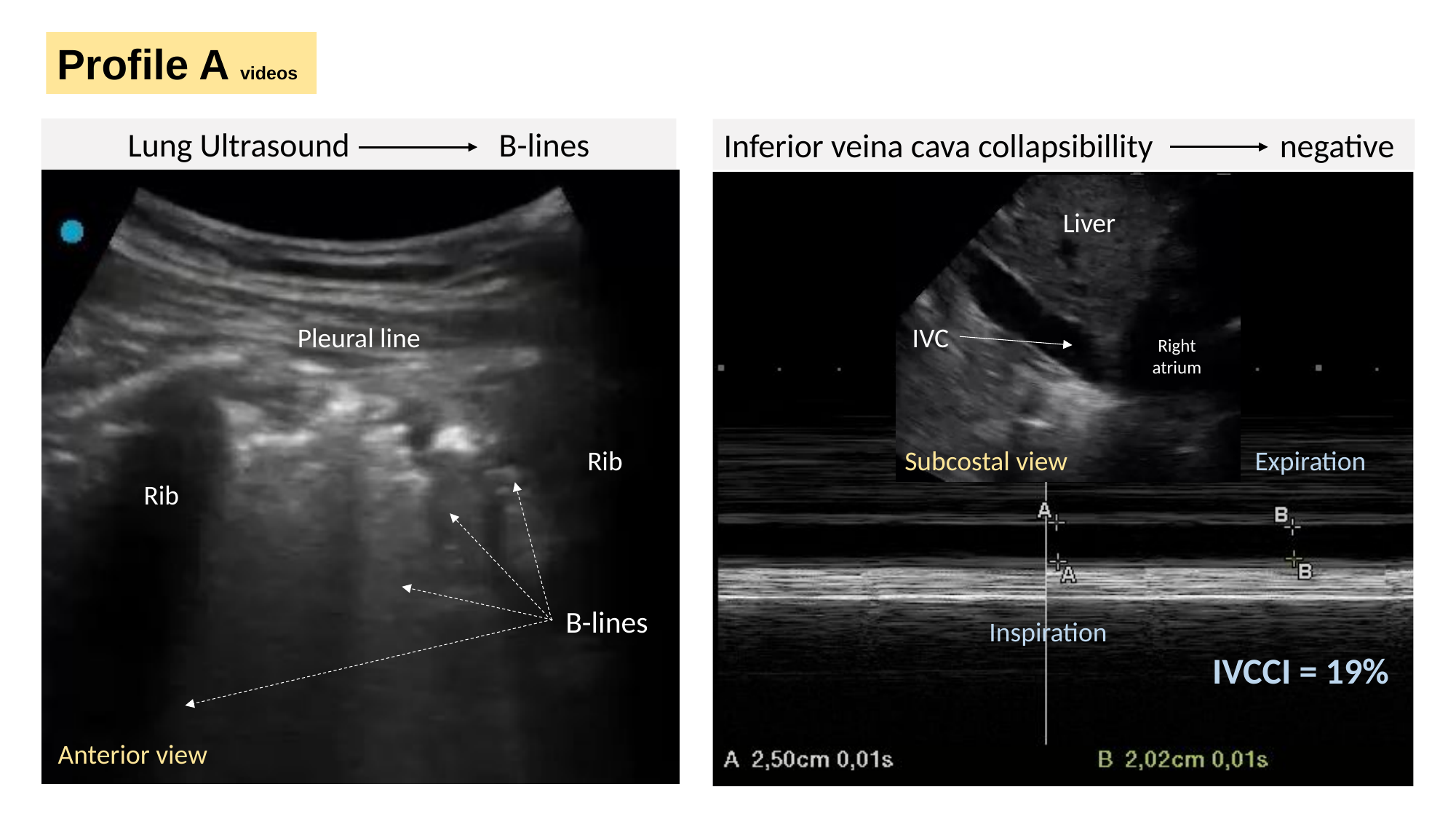

Profile A videos
Lung Ultrasound B-lines
Inferior veina cava collapsibillity negative
Liver
IVC
Pleural line
Right atrium
Expiration
Subcostal view
Rib
Rib
B-lines
Inspiration
IVCCI = 19%
Anterior view
