# Supplementary material for: Ultrasound-based clinical profiles for predicting the risk of intradialytic hypotension in critically ill patients on intermittent dialysis: a prospective observational study
Source: Crit Care. 2019 Dec 2;23:389. doi: 10.1186/s13054-019-2668-2 (PMC6889608; doi:10.1186/s13054-019-2668-2)
Supplement: Supplementary file 2 — Additional file 2. Patient with B lines < 14 and VCDi ≤11.5 mm.m− 2. [file 13054_2019_2668_MOESM2_ESM.pptx]

## Slide 1
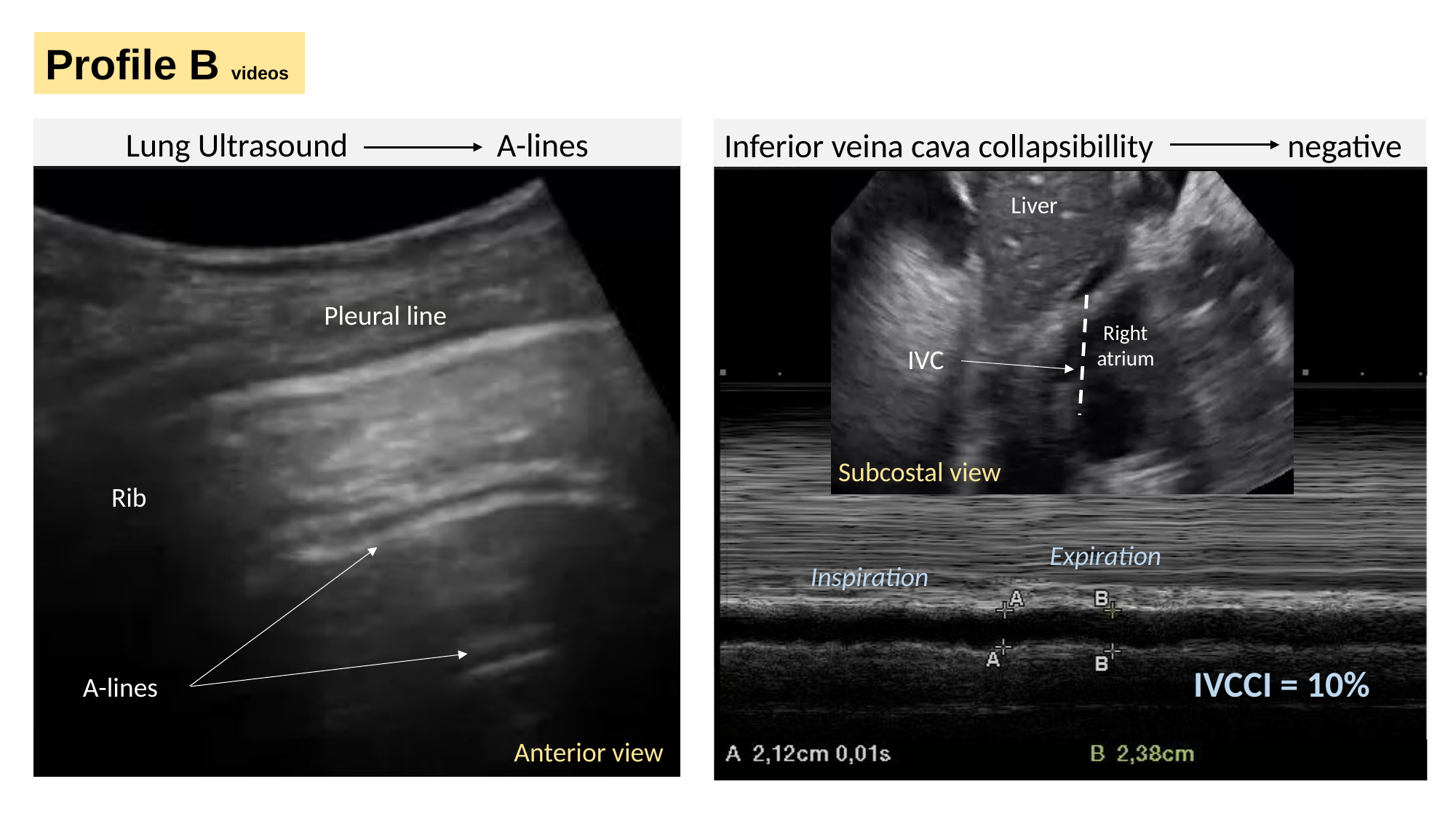

Profile B videos
Lung Ultrasound A-lines
Inferior veina cava collapsibillity negative
Expiration
Inspiration
IVCCI = 10%
Liver
Pleural line
Right atrium
IVC
Subcostal view
Rib
A-lines
Anterior view
