# Supplementary material for: Ultrasound-based clinical profiles for predicting the risk of intradialytic hypotension in critically ill patients on intermittent dialysis: a prospective observational study
Source: Crit Care. 2019 Dec 2;23:389. doi: 10.1186/s13054-019-2668-2 (PMC6889608; doi:10.1186/s13054-019-2668-2)
Supplement: Supplementary file 3 — Additional file 3. Patient with B lines > 14 and VCDi ≤11.5 mm.m− 2. [file 13054_2019_2668_MOESM3_ESM.pptx]

## Slide 1
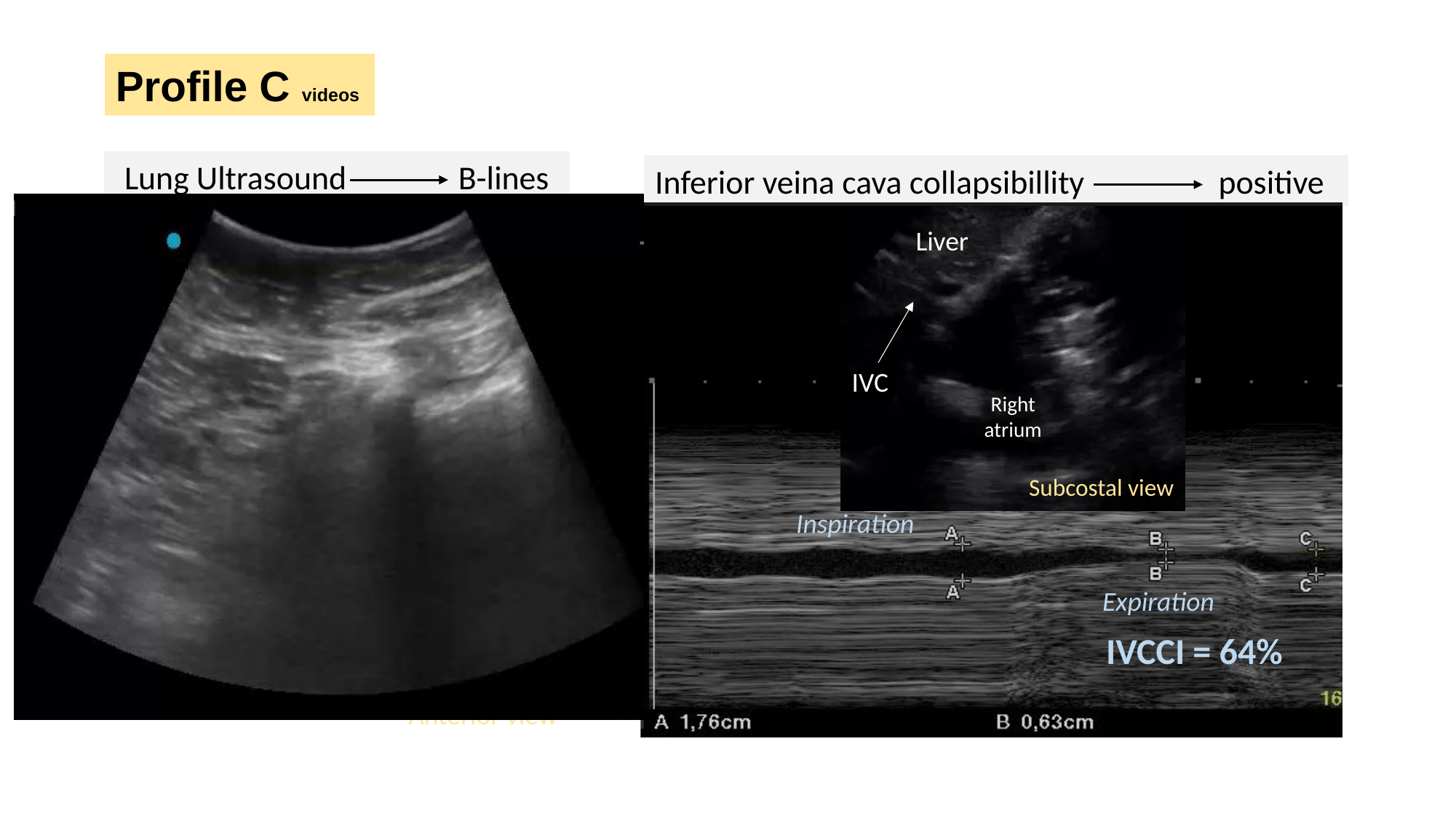

Profile C videos
Liver
Lung Ultrasound B-lines
Inferior veina cava collapsibillity positive
Inspiration
Expiration
IVCCI = 64%
Liver
Pleural line
Rib
Rib
IVC
Subcostal view
Right atrium
B-lines
Subcostal view
Anterior view
