# Supplementary material for: Ultrasound-based clinical profiles for predicting the risk of intradialytic hypotension in critically ill patients on intermittent dialysis: a prospective observational study
Source: Crit Care. 2019 Dec 2;23:389. doi: 10.1186/s13054-019-2668-2 (PMC6889608; doi:10.1186/s13054-019-2668-2)
Supplement: Supplementary file 4 — Additional file 4. Patient with B lines < 14 and VCDi > 11.5 mm.m− 2. [file 13054_2019_2668_MOESM4_ESM.pptx]

## Slide 1
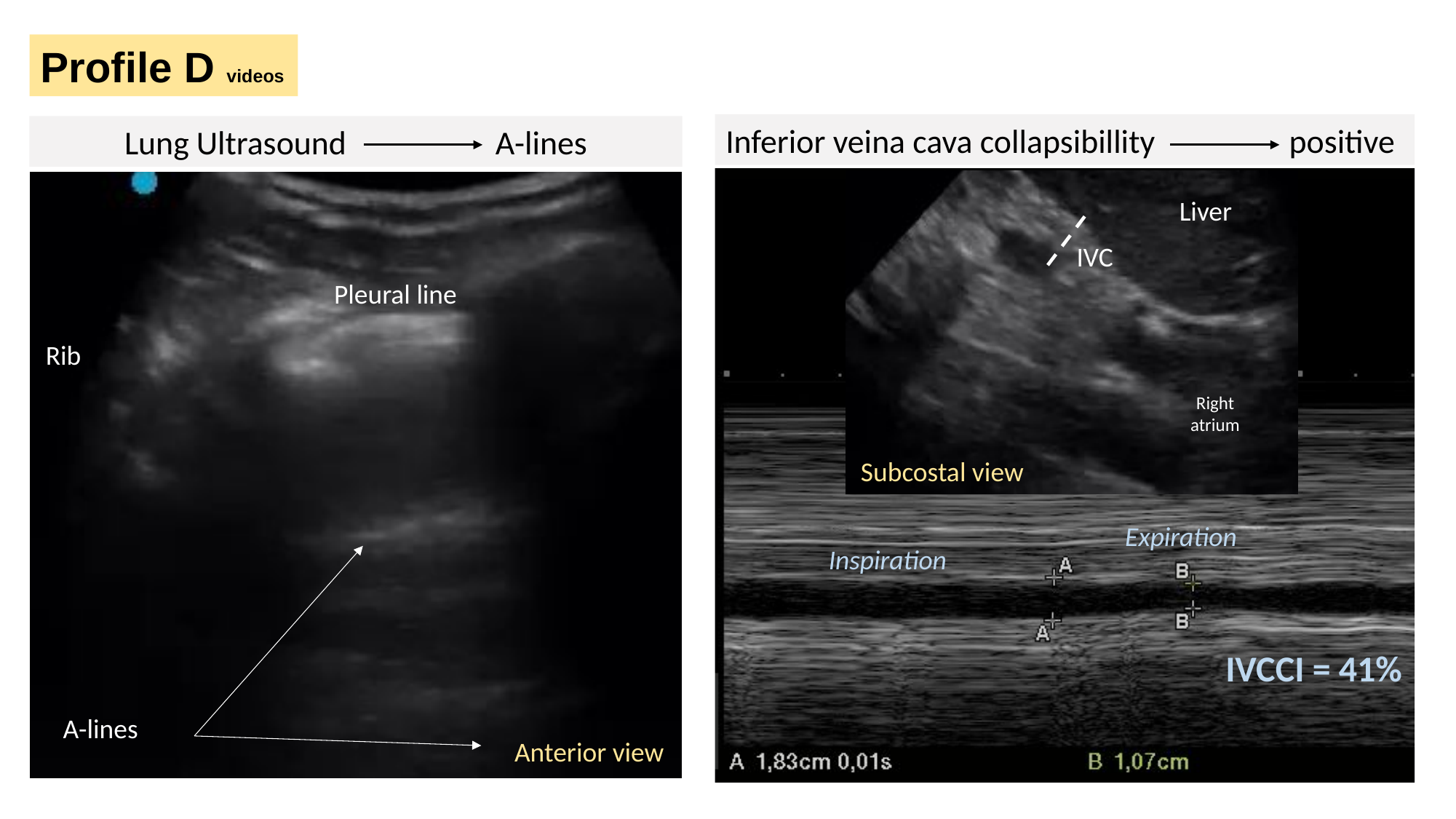

Profile D videos
Inferior veina cava collapsibillity positive
Lung Ultrasound A-lines
Expiration
Inspiration
IVCCI = 41%
Liver
IVC
Pleural line
Rib
Right atrium
Subcostal view
A-lines
Anterior view
