# Supplementary material for: Ultrasound-based clinical profiles for predicting the risk of intradialytic hypotension in critically ill patients on intermittent dialysis: a prospective observational study
Source: Crit Care. 2019 Dec 2;23:389. doi: 10.1186/s13054-019-2668-2 (PMC6889608; doi:10.1186/s13054-019-2668-2)
Supplement: Supplementary file 5 — Additional file 5. Ultrasound procedures and classifications [file 13054_2019_2668_MOESM5_ESM.docx]

Additional file 5

**Ultrasound Procedures and Classifications**

Vena Cava Collapsibility Measurement**:** A Sonosite M-turbo (Bothell, WA, USA) ultrasound machine was used to evaluate the vena cava collapsibility. The patients underwent a bedside ultrasound examination immediately before their dialysis sessions, in the supine position with 30° inclination. The probe was placed in the subxiphoid region and a sagittal view of the inferior vena cava (IVC) was obtained by B-mode ultrasonography, just below the diaphragm in the hepatic segment. After visualizing the IVC, a loop was acquired while taking care to maximize the IVC diameter throughout the respiratory cycle, by adjusting the angle between the transducer and the body. Images were frozen and scrolled to find the maximal IVC diameter (IVC_max_) during passive expiration, immediately after the hepatic vein or within 2.5 cm of the junction of the IVC with the right atrium, when the hepatic vein could not be visualized. Then, the minimum IVC diameter (IVC_min_) was measured during inspiration. The indexed IVC expiratory diameter (VCDi) was calculated by dividing the IVC_max_ (in mm) by the body surface area (in m^2^). The IVC collapsibility index (IVCCI; in %) was calculated by using the following standard formula: [(IVC_max_− IVC_min_)/IVC_max_] × 100. Volemic status was classified as hypervolemia if characterized by an IVCCI < 40% and/or VCDi > 11.5 mm.m^-2^ .

B lines determination**:** The scanning protocol consisted of scanning in the parasternal, midclavicular, anterior axillary, and midaxillary positions of the second to fifth intercostal spaces on the right side and second to fourth spaces on the left side for a total of 28 positions per complete examination. B-lines were defined as an echogenic, coherent, dynamic, wedge-shaped signal, with a narrow origin in the near field of the image, arising from the pleural line and extending to the edge of the screen. Multiple spaced B lines were defined as three or more B-lines in a given lung region and considered as representative of interstitial lung congestion. Coalescent B lines were considered representative of alveolar lung congestion. The sum of B lines in anterior and lateral lung regions (coalescent B lines were counted as 10 B lines) produced a lung ultrasound score reflecting the extent of pulmonary congestion. Pulmonary congestion was diagnosed if there was more than 14 B lines.
